# Editorial: Nutritional epidemiology: advances in the analysis of healthy and sustainable dietary patterns

**DOI:** 10.3389/fnut.2026.1773578

**Published:** 2026-02-10

**Authors:** Gladys Morales, Sebastián Cofre, Solange Parra-Soto, Israel Ríos-Castillo

**Affiliations:** 1 Department of Public Health, Universidad de La Frontera, Temuco, Chile; 2 Faculty of Health Sciences, School of Nutrition and Dietetics, Universidad Católica del Maule, Talca, Chile; 3 Department of Nutrition and Public Health, Faculty Health Science and Food, Universidad del Bío-Bío, Chillán, Chile; 4 Food and Agriculture Organization of the United Nations, Subregional Office for Mesoamerica, Panama City, Panama

**Keywords:** dietary pattern, epidemiology, food system, index, nutritional epidemiology, sustainability

There is growing interest in healthy, sustainable dietary patterns and food system transformation due to the intersection of human health, social equity, and environmental sustainability. Healthy diets not only prevent chronic diseases but also modulate mechanisms of biological aging and metabolic function through pathways involving lipid metabolism, body composition, and systemic inflammation. The studies presented in this Research Topic address these dimensions from a multidimensional perspective, integrating metabolic and microbiome biomarkers with ecological and social indices that assess the sustainability of food systems.

Chen et al. demonstrated that higher adherence to the *Planetary Health Diet Index (PHDI)* was associated with a lower risk of sarcopenia. This relationship is partially mediated by an improved lipid profile, highlighting the potential of sustainable diets to preserve muscle mass and support metabolic health across the lifespan. Similarly, Wei et al. showed that adherence to healthy dietary patterns, such as AHEI, DASH, or MEDI, was associated with slower biological aging and a lower risk of kidney stones, underscoring that overall diet quality can directly influence physiological processes related to longevity.

Furthermore, Liu et al. and Long et al. presented compelling evidence that the gut microbiota serves as a key mediator between diet, metabolism, and cardiovascular health, drawing on the newly developed *Dietary Index for Gut Microbiota (DI-GM)*. Both studies reported that greater microbial diversity, reflecting better dietary quality, was inversely associated with metabolic syndrome and cardiovascular-kidney-metabolic (CKM) syndrome, with BMI emerging as an essential mediator. These findings position the DI-GM as an innovative dietary biomarker that integrates metabolic health and dietary sustainability by modulating the gut microbiota.

The environmental dimension of dietary sustainability also occupies a central place in this Research Topic. Conrad et al. modeled the ecological impacts of replacing different protein sources among U.S. adults. They reported that replacing beef with pork markedly reduced greenhouse gas emissions and land use, whereas substituting legumes or poultry with pork increased environmental impacts. Recent evidence highlights the critical role of aquatic foods, particularly fish and seafood, in advancing both human and planetary health (4). These findings reveal that sustainability outcomes depend on the type of food substitution, emphasizing the need for more precise food policies that balance health and environmental goals.

Complementarily, Contreras-Núñez et al.. developed the *Sustainability Diet Index (S-DI)*, a multicriteria decision analysis tool applied to institutional menus that integrates the nutritional, environmental, and social dimensions of food choices. This tool demonstrated that menus containing local ingredients and a lower proportion of animal-based foods achieved the highest sustainability scores. The S-DI thus emerges as a practical framework for translating sustainability principles into the culinary field and collective food services. In addition, the updated EAT–Lancet Commission on healthy, sustainable, and just food systems report says that healthy and sustainable diets should be predominantly plant-based, but with flexibility to include moderate amounts of animal-sourced foods, such as fish, dairy, and eggs, according to cultural and nutritional contexts (1).

The social dimension of sustainability is illustrated in the study by Cisneros-Vásquez et al., who reported that food insecurity persists even in high-income countries, disproportionately affecting adolescents from immigrant or low-education families. This evidence underscores the urgent need to integrate food justice and equitable access within the global agenda of sustainable nutrition. Research in nutritional epidemiology must incorporate new approaches, such as qualitative or mixed-method designs, that allow for a more multidimensional understanding of diet and sustainability (2).

Finally, the meta-analysis conducted by Mo et al. reaffirmed the association between plant-based dietary patterns and longevity, showing that greater adherence to healthy plant-based diets was associated with lower total and cardiovascular mortality. In contrast, unhealthy plant-based diets increased mortality risk.

The studies included in this Research Topic demonstrate significant progress in developing integrative dietary indices that quantify the relationships among diet quality, metabolic wellbeing, and environmental sustainability. The PHDI operationalizes the concept of the planetary health diet by linking nutritional adequacy with ecological impact. The DI-GM, introduces a microbiota-centered perspective as a novel component for evaluating diet quality. Finally, the S-DI extends this approach to the culinary and institutional management sphere, integrating practical sustainability metrics into food service systems.

Future research should move beyond observational studies and toward public-level interventions capable of influencing population behavior. Importantly, this does not imply relying exclusively on randomized controlled trials. Pragmatic trials and quasi-experimental designs that draw on real-world observational data are equally necessary. Causal inference approaches can strengthen these designs by improving estimates of the feasibility, effectiveness, and scalability of modifying food environments, ensuring that sustainability is incorporated from study design to measurable outcomes.

It is essential to emphasize the importance of integrating nutrition and sustainability into public health policy. Together, these studies demonstrate that healthy and sustainable diets are fundamental for improving population health, reducing environmental degradation, and promoting social equity. Indices such as the PHDI, DI-GM, and S-DI help support the development of evidence-based public policies, as well as educational programs and transdisciplinary research aligned with the Sustainable Development Goals (3). The adoption of approaches such as those presented in this Research Topic may help drive food system transitions that are just, resilient, and health-promoting. By linking biological aging, metabolic regulation, and planetary boundaries, this Research Topic outlines a holistic pathway toward nutrition for planetary health, where dietary choices support the wellbeing of both people and the planet.

## References

[1] Rockström J Thilsted SH Willett WC Gordon LJ Herrero M Hicks CC . The EAT-Lancet Commission on healthy, sustainable, and just food systems. Lancet. (2025) 406:1625–700. doi: 10.1016/S0140-6736(25)01201-2 41046857

[2] Guillaumie L Boiral O Baghdadli A Mercille G. Integrating sustainable nutrition into health-related institutions: a systematic review of the literature. Can J Public Health. (2020) 111:845–61. doi: 10.17269/s41997-020-00394-3 32959328 PMC7728986

[3] United Nations. Transforming Our World: The 2030 Agenda for Sustainable Development. New York, NY: United Nations (2015). Available online at: https://sdgs.un.org/2030agenda (Accessed December 1, 2025).

[4] Jones AR Alleway HK McAfee D Reis-Santos P Theuerkauf SJ Jones RC. Climate-friendly seafood: the potential for emissions reduction and carbon capture in marine aquaculture. Biosci. (2022) 72:123–43. doi: 10.1093/biosci/biab126 35145350 PMC8824708

